# Oral Ibrexafungerp for Vulvovaginal Candidiasis Treatment: An Analysis of VANISH 303 and VANISH 306

**DOI:** 10.1089/jwh.2022.0132

**Published:** 2023-02-08

**Authors:** Oluwatosin Goje, Ryan Sobel, Paul Nyirjesy, Steven R. Goldstein, Mark Spitzer, Brooke Faught, Shelagh Larson, Thomas King, Nkechi E. Azie, David Angulo, Jack D. Sobel

**Affiliations:** ^1^ Department of Obstetrics and Gynecology, Cleveland Clinic Foundation, Case Western Reserve University, Cleveland, Ohio, USA.; ^2^ Department of Obstetrics and Gynecology, Jefferson Vulvovaginal Health Center, Sidney Kimmel Medical College, Thomas Jefferson University, Philadelphia, Pennsylvania, USA.; ^3^ Department of Obstetrics and Gynecology, NYU Grossman School of Medicine, New York, New York, USA.; ^4^ Center for Colposcopy, New Hyde Park, New York, USA.; ^5^ Department of Obstetrics and Gynecology, Donald and Barbara Zucker School of Medicine at Hofstra/Northwell, Manhasset, New York, USA.; ^6^ Women's Institute for Sexual Health, Division of Urology Associates, PC, Nashville, Tennessee, USA.; ^7^ Department of Women and Infants, Acclaim Physician Group-Fort Worth, Fort Worth, Texas, USA.; ^8^ Department of Medical Affairs, SCYNEXIS, Inc., Jersey City, New Jersey, USA.; ^9^ Department of Clinical Development and Medical Affairs, Wayne State University, Detroit, Michigan, USA.; ^10^ Department of Research and Development, SCYNEXIS, Inc., Jersey City, New Jersey, USA.; ^11^ Infectious Diseases, Department of Internal Medicine, Wayne State University, Detroit, Michigan, USA.

**Keywords:** antifungal, ibrexafungerp, vulvovaginal candidiasis, placebo, *Candida albicans*

## Abstract

**Background:**

Ibrexafungerp is a novel antifungal treatment for acute vulvovaginal candidiasis (VVC). Using pooled data from two phase three studies (VANISH 303 and 306) in the treatment of acute VVC, this analysis sought to determine the effectiveness of ibrexafungerp in various patient subgroups that may impact outcomes.

**Materials and Methods:**

Data from VANISH 303 (NCT03734991) and VANISH 306 (NCT03987620) evaluating ibrexafungerp 300 mg twice daily (BID) for 1 day versus placebo, were pooled and analyzed to determine clinical cure rate, clinical improvement, and mycological cure at the test-of-cure visit (day 11 ± 3) and symptom resolution at the follow-up visit (day 25 ± 4) in the overall population. Patient subgroups analyzed included race, body mass index (BMI), baseline vulvovaginal signs and symptoms (VSS) score, and *Candida* species.

**Results:**

At the test-of-cure visit, patients receiving ibrexafungerp, compared with those who received placebo, had significantly higher rates of clinical cure (56.9% [214/376 patients] vs. 35.7% [65/182 patients]), clinical improvement (68.4% [257/376 patients] vs. 45.1% [82/182 patients]), and mycological cure (54.0% [203/376 patients] vs. 24.2% [44/182 patients]; all *p* < 0.0001). At the follow-up visit, patients receiving ibrexafungerp had sustained responses with higher symptom resolution rates (66.8% [251/376 patients]) versus placebo (48.4% [88/182 patients]; *p* < 0.0001). Race, BMI, baseline VSS score (including VSS severity score 13–18), and *Candida* species infection did not adversely affect clinical cure rates. Safety analysis results were consistent with the individual studies.

**Conclusions:**

Ibrexafungerp provides a safe and well-tolerated first-in-class fungicidal, 1-day oral treatment for patients with acute VVC, the first new therapy in >20 years. Clinical Trial Registration Number: NCT03734991.

## Introduction

Vulvovaginal candidiasis (VVC), the second most common cause of vaginitis, negatively impacts the quality of life of affected women.^1–6^ It has been associated with emotional and psychosocial distress, loss of productivity, and an economic burden on outpatient health care systems.^1,7,8^ For more than 20 years, treatment options for acute VVC had been limited to short courses of topical azole formulations and oral fluconazole.^2,4–6^ In June 2021, the U.S. Food and Drug Administration (FDA) approved the first oral nonazole therapy for the treatment of acute VVC—ibrexafungerp, a first-in-class triterpenoid antifungal. Ibrexafungerp exerts fungicidal activity against most *Candida* isolates by targeting the glucan synthase enzyme responsible for producing glucan polymers, a key component of the fungal cell wall, resulting in fungal cell death.^9–11^ In preclinical studies, ibrexafungerp has demonstrated vaginal tissue concentrations two- to ninefold higher than plasma and retention of its activity in a low pH (4.5) environment, consistent with a vaginal pH commonly seen in patients with VVC.^4,12–14^

Approval of ibrexafungerp for the treatment of acute VVC was based, in part, on results from two identical phase three studies (VANISH 303 and VANISH 306).^15,16^ These studies reported oral ibrexafungerp 300 mg twice daily (BID) for 1 day to be well tolerated and statistically superior to placebo in efficacy end points evaluated.^15,16^ The design of both studies was based on FDA guidance released in 2019 for the development of drugs for the treatment of VVC, which included recommendations for trial design, efficacy end point, and the choice of comparators (*e.g.*, placebo vs. an active comparator).^15–17^

The definition of efficacy end points in the VANISH studies differed from those previously reported in VVC studies, with one of the most significant differences being the definition of clinical cure. In previous studies, clinical cure has been reported as a resolution of all signs and symptoms, with a total severity score ≤2 (vulvovaginal signs and symptoms [VSS] ≤2), whereas clinical cure in the VANISH studies was defined as the absence of all VSS of VVC (VSS score = 0).^15–20^ The timing of response assessments also differs from some previously published studies, which can impact reported outcomes.^17,18,20,21^ Although these guidelines will help standardize VVC studies moving forward, they make historical comparisons to previous studies difficult.

While the efficacy of ibrexafungerp in acute VVC has already been established in previous studies,^15,16,22^ we had an opportunity to further critically analyze the data in the two phase three VANISH studies,^15,16^ which were not feasible to evaluate in the previous individual publications, to determine the effectiveness of ibrexafungerp in various patient subgroups that may impact outcomes as demonstrated in previous studies (*e.g.*, baseline VSS score^23^ and *Candida* species^24^). Given the controversy as to whether body mass index (BMI) plays a role in acute VVC, we evaluated the effectiveness of ibrexafungerp in patients with various BMIs in this pooled analysis. We also sought to evaluate the impact of race on ibrexafungerp effectiveness, as an increased incidence of acute VVC has been reported in Black women compared with White women.^25,26^

## Materials and Methods

This analysis pooled data from VANISH 303 (NCT03734991) and VANISH 306 (NCT03987620). Methods, overall efficacy, and tolerability and safety results for these two studies have been published previously.^15,16^ These studies compared oral ibrexafungerp with placebo in postmenarchal female patients aged ≥12 years with acute VVC and a VSS score ≥4 at baseline, and at least two signs or symptoms having a score of ≥2. Patients rated symptoms of burning, itching, and irritation and investigators rated the signs of edema, erythema, and excoriation/fissures on a scale of severity (0 = absent; 1 = mild; 2 = moderate; 3 = severe) to calculate a total composite score (range, 0–18). Other inclusion criteria included a normal vaginal pH (≤4.5), a positive result on microscopic examination with 10% potassium hydroxide revealing yeast forms, and contraceptive use in patients of reproductive potential.

Exclusion criteria included patients pregnant, lactating, or likely to become pregnant; any condition that may have interfered with the diagnosis or evaluation of response to therapy, including mixed infections; the use of systemic and/or topical (vaginal) antifungal treatment products within 28 days of baseline; patients with known HIV infection and/or receiving treatment that could compromise their immune response; and patients with history of or active cervical or vaginal cancer. The only difference between the two studies was that VANISH 303 included only sites in the United States, whereas VANISH 306 included sites in both the United States and Bulgaria.

Both studies were conducted in accordance with the principles of the Declaration of Helsinki. Before study initiation, each study site obtained institutional review board approval. All patients provided written consent for study participation. Patients were randomized at a 2:1 ratio to receive either ibrexafungerp 300 mg BID for 1 day or matching placebo. Randomization was stratified according to diabetes mellitus diagnosis (yes or no). All site and sponsor personnel were blinded to treatment assignments except for a team member who was responsible for drug distribution logistics. To maintain blinding, appearance was similar between active and placebo dose forms, both of which were provided by SCYNEXIS, Inc. (Jersey City, NJ, USA).

The objective of these pooled analyses was to evaluate the efficacy of oral ibrexafungerp compared with placebo in patients with acute VVC, with efficacy based on the clinical cure rate at the test-of-cure visit using the pooled data from VANISH 303 and VANISH 306.^15,16^ Additional end points at the test-of-cure visit included clinical improvement and mycological cure (mycological eradication). Symptom resolution was evaluated at the follow-up visit. Definitions of efficacy end points are provided in Table 1. Subgroup analyses were conducted for clinical cure by race, BMI, baseline VSS score, and *Candida* species. Adverse events were coded using the Medical Dictionary for Regulatory Activities (MedRA; version 21.1). Efficacy analyses used the modified intention to treat (mITT) population, which consisted of all patients randomly assigned to a treatment group with a positive culture for *Candida* species at baseline who received ≥1 dose of the study drug. Safety analyses used the safety population, which included all patients randomly assigned to a treatment group who received ≥1 dose of the study drug and had ≥1 postbaseline evaluation.

**Table 1.** Efficacy End Point Definitions

End point	Definition
Clinical cure	Complete resolution of signs and symptoms of vulvovaginal infection without need for further antifungal treatment or topical vaginal drug therapy for the treatment of vulvovaginal irritation (burning)/pruritus before or at the test-of-cure visit. VSS score = 0 at the test-of-cure visit.
Clinical improvement	Partial or complete resolution of signs and symptoms of vulvovaginal infection with total composite score ≤1 at the test-of-cure visit without need for further antifungal treatment or topical drug therapy for the treatment of vulvovaginal irritation (burning)/pruritis before or at the test-of-cure visit. VSS score ≤1 at the test-of-cure visit.
Complete resolution of symptoms at the follow-up visit	Complete resolution of symptoms in patients at the follow-up visit. Symptom score = 0 at the follow-up visit.
Mycological cure	Negative culture for *Candida* species without need for further antifungal treatment at the test-of-cure visit.

VSS, vulvovaginal signs and symptoms.

Statistical analysis for overall efficacy outcomes was performed using SAS software version 9.4 (SAS Institute Inc., Cary, NC, USA). The Cochran–Mantel–Haenszel method was used to adjust for country and diabetes mellitus diagnosis and to assess the statistical significance of a difference between treatment groups. The *p*-value, relative risk (RR), and 95% confidence interval [CI] are presented. Categorical data were summarized using the patient count and percentage. A patient was considered a nonresponder if she did not meet the clinical response criteria for categorical responses or was missing categorical response data at the specific visit. Per study protocol, randomized patients without ≥1 postbaseline observation were also defined as nonresponders, such that patients who had a missing value at the test-of-cure or follow-up visit were deemed to be treatment failures.

SCYNEXIS, Inc. sponsored this study and was responsible for working with authors in the development of the protocols; in the collection, analysis, and interpretation of study data; in writing of the clinical study report; and in the decision to submit the article for publication. The authors' personal interests, financial or nonfinancial, relating to this research and its publication have been disclosed.

## Results

**FIG. 1.**
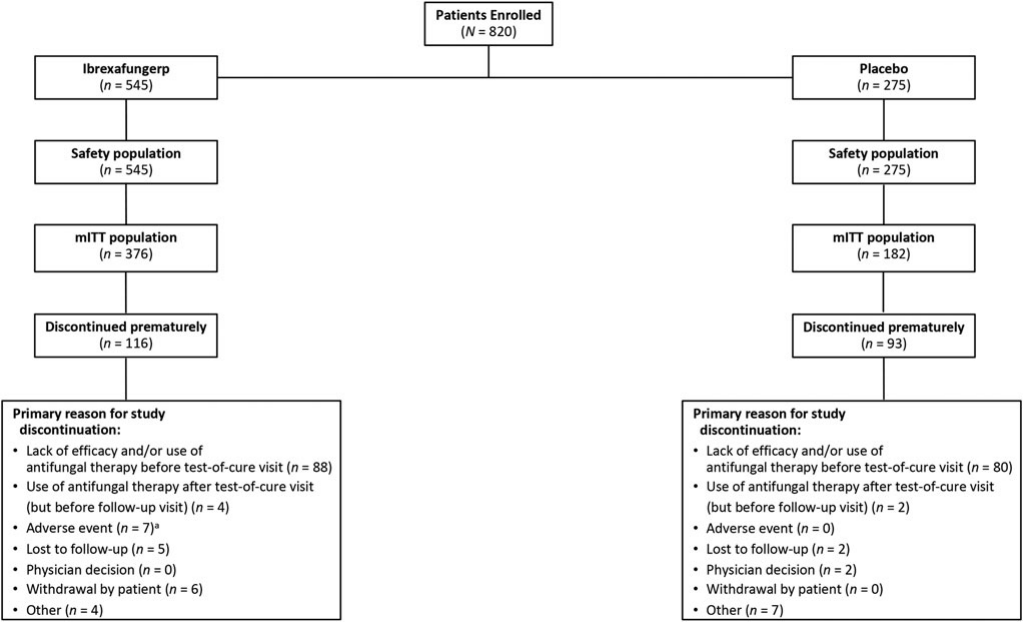
Patient disposition of the pooled analysis of the VANISH 303 and VANISH 306 studies. ^a^Five patients experienced a TEAE not related to ibrexafungerp that led to study discontinuation (bacterial vaginosis, *n* = 4; diabetes mellitus, *n* = 1). mITT, modified intention to treat; TEAE, treatment-emergent adverse event.

A total of 376 patients from the United States were randomly assigned to receive ibrexafungerp (*n* = 249) or placebo (*n* = 127) in the VANISH 303 study; of these, 286 patients were included in the mITT population (ibrexafungerp, *n* = 188; placebo, *n* = 98). In the VANISH 306 study, 449 patients from Bulgaria or the United States were randomly assigned to receive ibrexafungerp (*n* = 298) or placebo (*n* = 151); of these, 272 patients were included in the mITT population (ibrexafungerp, *n* = 188; placebo, *n* = 84). The pooled mITT population included 376 patients who received ibrexafungerp and 182 patients who received placebo. The pooled safety population included 545 patients who received ibrexafungerp and 275 who received placebo (Fig. 1).

Demographics and baseline characteristics of the mITT population are presented in Table 2. Overall, baseline characteristics of the ibrexafungerp and placebo groups were well balanced. Fewer than 8% of patients in each treatment group had diabetes mellitus, and >90% of patients had a baseline severity score of ≥7. All patients had a positive culture for ≥1 *Candid*a species at baseline, with >85% of patients testing positive for *Candida albicans* in both treatment groups. Similar baseline severity scores were observed in women with baseline non-*albicans Candida* species, with scores ranging from 4 to 18; severity scores ≥7 occurred in 89.9% of these patients. As per previous reports,^15,16^ no fluconazole-resistant isolates of *C albicans* were identified at baseline using Clinical and Laboratory Standards Institute M27-A3 guidelines (VANISH 303 and VANISH 306) and European Committee on Antimicrobial Susceptibility Testing E.DEF 7.3.1 methods (VANISH 306); at the test-of-cure visit, there was no notable change in susceptibility following ibrexafungerp exposure.

**Table 2.** Patient Characteristics and Demographics (Modified Intention to Treat Population)

	Ibrexafungerp 300 mg BID for 1 day (*N* = 376)	Placebo (*N* = 182)
Age, years		
Mean (SD)	33.6 (10.33)	34.8 (11.59)
Median (min, max)	32.0 (18, 67)	32.0 (17, 66)
Age category, years, *n* (%)		
<65	374 (99.5)	179 (98.4)
≥65	2 (0.5)	3 (1.6)
BMI, kg/m^2^, *n* (%)		
≤35	311 (82.7)	145 (79.7)
>35	65 (17.3)	37 (20.3)
Underweight (<18.5)	21 (5.6)	9 (4.9)
Normal (18.5–<25)	153 (40.7)	67 (36.8)
Overweight (25–<30)	81 (21.5)	39 (21.4)
Obese (30–<40)	91 (24.2)	45 (24.7)
Morbidly obese (≥40)	30 (8.0)	22 (12.1)
Race, *n* (%)		
White	256 (68.1)	122 (67.0)
Asian	4 (1.1)	0 (0.0)
Black	107 (28.5)	58 (31.9)
American Indian or Alaska Native	3 (0.8)	0 (0.0)
Other^a^	6 (1.6)	2 (1.1)
Regional subgroups, *n* (%)		
United States	254 (67.6)	134 (73.6)
Bulgaria	122 (32.4)	48 (26.4)
Diabetes		
Yes	27 (7.2)	13 (7.1)
No	349 (92.8)	169 (92.9)
Fungal pathogens,^b^ *n* (%)		
*C albicans*	322 (85.6)	156 (85.7)
*C glabrata*	31 (8.2)	19 (10.4)
Other^c^	23 (6.1)	7 (3.8)
Baseline VSS score, *n* (%)		
<7	22 (5.9)	15 (8.2)
≥7	354 (94.1)	167 (91.8)
4–7	59 (15.7)	43 (23.6)
8–12	253 (67.3)	113 (62.1)
13–18	64 (17.0)	26 (14.3)

^a^ Included multiracial patients and patients in whom a race was not identified.

^b^ Women may have had more than one *Candida* species identified at baseline and can be counted once at each species level.

^c^ Other species in the ibrexafungerp versus placebo groups, respectively, included *C. dubliniensis* (2 vs. 1); *C. inconspicua* (1 vs. 0); *C. kefyr* (3 vs. 1); *C. krusei* (2 vs. 1); *C. lusitaniae* (2 vs. 1); *C. norvegensis* (1 vs. 0); *C. parapsilosis* (4 vs. 0); *C. tropicalis* (7 vs. 3); and *Saccharomyces* species (1 vs. 0).

BID, twice daily; BMI, body mass index; max, maximum; min, minimum; mITT, modified intention to treat; VSS, vulvovaginal signs and symptoms.

### Overall results

**FIG. 2.**
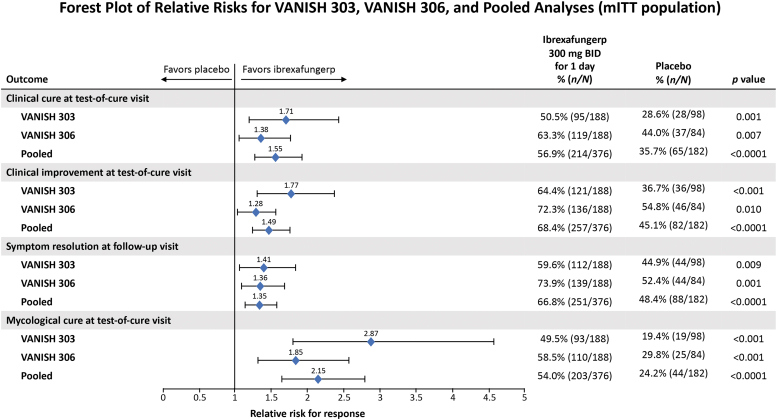
Efficacy outcomes for the VANISH 303, VANISH 306, and pooled analysis studies. BID, twice daily; mITT, modified intention to treat.

Ibrexafungerp treatment resulted in similar improvements in all efficacy end points, all of which were statistically superior to placebo in VANISH 303, VANISH 306, and in the pooled analysis (Fig. 2). In the pooled analysis, the clinical cure rate (VSS score = 0) at the test-of-cure visit was significantly higher in patients receiving ibrexafungerp than those receiving placebo (56.9% vs. 35.7%; RR, 1.55; 95% CI, 1.26–1.91; *p* < 0.0001). Patients receiving ibrexafungerp also experienced higher rates of clinical improvement (VSS score ≤1) at the test-of-cure visit compared with placebo (68.4% vs. 45.1%; RR, 1.49; 95% CI, 1.26–1.77; *p* < 0.0001). In previous studies,^18,19,24^ clinical cure has been defined as VSS score ≤2. In *post hoc* analyses of VANISH 303 and VANISH 306, clinical cure was evaluated using VSS score ≤2 and yielded a pooled rate of 72.6% (273 of 376 patients) for ibrexafungerp patients.

The percentage of patients with complete symptom resolution at the follow-up visit (symptom score = 0 without having received rescue antifungal treatment regardless of having achieved a clinical cure at the test-of-cure visit) was also significantly higher with ibrexafungerp (66.8% vs. 48.4%; RR, 1.35; 95% CI, 1.15–1.59; *p* < 0.0001). The mycological cure rate (negative culture for *Candida* species) at the test-of-cure visit was also significantly higher with ibrexafungerp than with placebo (54.0% vs. 24.2%; RR, 2.15; 95% CI, 1.65–2.80; *p* < 0.0001).

In the pooled analysis, patients with vulvovaginal candidiasis (caused predominantly by baseline *C albicans* infections) reported significant improvement in all efficacy end points following treatment with ibrexafungerp compared with placebo. These findings are consistent with those of VANISH 303 and VANISH 306.

### Subgroup analyses

**FIG. 3.**
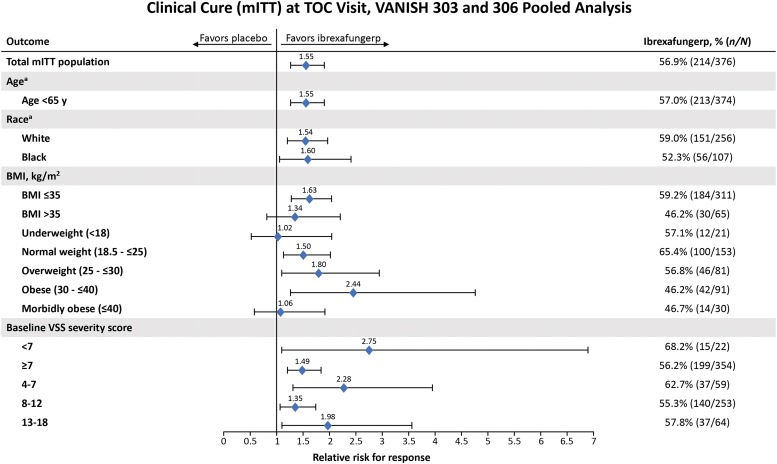
Clinical cure rates at the test-of-cure visit in patient subgroups in pooled analyses of VANISH 303 and VANISH 306 patients (mITT population). ^a^Insufficient number of patients to analyze ≥65 years of age, Asian, and Race (other) subgroups. BMI, body mass index; mITT, modified intention to treat; TOC, test-of-cure; VSS, vulvovaginal signs and symptoms.

Subgroup analyses of clinical cure at the test-of-cure visit were conducted for the following groups: race, BMI, baseline VSS score, and *Candida* species. Because only five patients were ≥65 years of age, an analysis of clinical cure by age could not be performed. Analyses of the remaining subgroups showed no influence on clinical cure rates (Fig. 3). In our analysis, 94.1% (354 of 376) of patients receiving ibrexafungerp had severe VVC (VSS score ≥7) at baseline and had a clinical cure rate (56.2%) similar to the overall population (56.9%). Clinical cure rates remained unchanged with increasing VSS severity score—55.3% in patients with baseline VSS scores of 8–12 and 57.8% in patients with baseline VSS scores of 13–18. In the *Candida* species groups, more than 85% of women in the mITT tested positive for *C albicans*. Clinical cure rates following ibrexafungerp therapy based on *Candida* species at baseline included 58.7% (189 of 322 patients) for *C albicans*, 46.3% (25 of 54 patients) for non-*albicans Candida* species, and 48.4% (15 of 31 patients) for *C glabrata*. Overall, outcomes were not adversely affected by race, weight, severity of infection, or underlying pathogen.

### Safety

In the pooled analysis, ibrexafungerp remained well tolerated with 26.1% (142 of 545 patients) treatment-related treatment-emergent adverse events (TEAEs) compared with 9.8% (27 of 275 patients) in the placebo group. The majority of adverse events reported were gastrointestinal related and mild-to-moderate in severity. The most commonly reported treatment-related TEAEs with ibrexafungerp (occurring in >2% of patients) compared with placebo included diarrhea (13.8% [75 of 545 patients] vs. 2.2% [6 of 275 patients]); nausea (8.4% [46 of 545 patients] vs. 2.5% [7 of 275 patients]); abdominal pain (3.1% [17 of 545 patients] vs. 0% [0 of 275 patients]); abdominal discomfort (2.6% [14 of 545 patients] vs. 0.7% [2 of 275 patients]); and dizziness (2.4% [13 of 545 patients] vs. 0.7% [2 of 275 patients]).

Seven of 545 women receiving ibrexafungerp discontinued the study or treatment due to an adverse event—vomiting (1), dizziness (1), bacterial vaginosis (4), and newly diagnosed diabetes mellitus (1). Two of these discontinuations were due to treatment-related TEAEs: vomiting of mild severity and dizziness of moderate severity. No patients in the placebo group discontinued due to a TEAE. No treatment-related serious adverse events or deaths were reported nor did any laboratory evaluations reveal any trends potentially associated with ibrexafungerp administration. Although patients of reproductive potential agreed to use an effective contraceptive method or remain abstinent through ≥10 days after completion of study therapy, three pregnancies were reported in the ibrexafungerp group; one pregnancy was electively terminated and two resulted in live births with no maternal or neonatal complications.

## Discussion

By pooling the data from the two phase three VANISH studies, the study population was doubled. To our knowledge, this has yielded the largest placebo-controlled data set in the treatment of acute VVC to date. The results of the pooled analyses confirm the overall high cure rates with ibrexafungerp 300 mg BID for 1 day seen in previous studies.^15,16^

Similar to the VANISH 303 and VANISH 306 results,^15,16^ ibrexafungerp demonstrated significant improvement compared with placebo in clinical cure (56.9% vs. 35.7%, respectively), clinical improvement (68.4% vs. 45.1%, respectively), and mycological cure rates (54.0% vs. 24.2%, respectively) at the test-of-cure visit (all *p* < 0.0001). At the follow-up visit, ∼14 days later, patients receiving ibrexafungerp had sustained responses with higher symptom resolution rates reported in 66.8% receiving ibrexafungerp regardless if they achieved a clinical cure at the test-of-cure visit compared with 48.4% of patients receiving placebo. Similar statistically significant efficacy outcomes were seen in patients with baseline *C albicans* infections, the species identified in 85%–90% of patients with VVC.^27,28^ In our study, rates of *C albicans* and non-*albicans Candida* infections were ≈85% and ≈14%, respectively, confirming that the majority of VVC infections are still predominantly caused by *C albicans*.^27,28^

To our knowledge, this pooled analysis is also the largest data set used to analyze treatment outcomes in subgroups of patients with acute VVC. In this subgroup analysis, race, BMI, and *C albicans* infection did not appear to influence clinical cure rates. Furthermore, clinical cure rates similar to the overall pooled cure rate were observed in patients regardless of baseline VSS score. This is important, as 94.1% (354 of 376) of patients receiving ibrexafungerp had severe VVC (VSS score ≥7), and we observed no change in clinical cure response rates with increasing severity of infection. Because fluconazole was not compared with ibrexafungerp in the VANISH studies, direct comparisons of the treatments and superiority of one treatment over the other cannot be concluded from our results.

In addition, historical comparisons of ibrexafungerp and fluconazole are difficult because of varying study methodologies. Several pivotal studies evaluating fluconazole in VVC reported 52%–65% of patients with severe VVC.^23,24^ In a previous study of fluconazole, baseline VSS scores ≥7 were associated with reduced response rates with single-dose fluconazole when treating severe VVC compared with mild disease (VSS score <7).^23^ In our pooled analysis, most patients receiving ibrexafungerp had baseline VSS scores ≥7 (*n* = 354) compared with <7 (*n* = 22), with the majority of patients receiving ibrexafungerp reporting a baseline VSS score between 8 and 12 (*n* = 253). Results from this pooled analysis demonstrate that patients with acute VVC, regardless of baseline severity score, can be treated effectively with the convenience of a 1-day dose of ibrexafungerp. In comparison, cases of severe VVC may respond with multiple-day dosing of fluconazole.^24^ For severe VVC, current guidelines recommend oral fluconazole every 3 days for a total of two to three doses.^4^

In a prior *post hoc* analysis, the efficacy of ibrexafungerp was not affected by Black race or BMI >35.^15^ In this pooled analysis, we confirm that race (White and Black) did not affect efficacy outcomes with ibrexafungerp. Analysis of ibrexafungerp in other races is warranted. In addition, BMI did not appear to be a predictor of response with ibrexafungerp based on the fluctuations in relative risk between the various BMI categories.

In the pooled analysis, patients with baseline infections of non-*albicans Candida* species had lower clinical cure rates than did patients with *C albicans* infections. Of the non-*albicans Candida* species, *C glabrata* was the most common species. Patients with *C glabrata* infections also had lower clinical cure rates compared with patients with *C albicans* infections. Despite pooling patient data from the two VANISH studies,^15,16^ the sample size for patients with non-*albicans Candida* infections remained small, although proportional to epidemiologically reported rates,^27^ thereby limiting the interpretation of these results. Nevertheless, continuing evaluation of ibrexafungerp in patients with non-*albicans Candida* species is warranted. Lower efficacy rates have been previously reported in women treated with fluconazole for non-*albicans Candida* infections compared with *C albicans* infection.^24^

Safety and tolerability results in this pooled analysis were similar to those reported in the VANISH 303 and VANISH 306 studies,^15,16^ with mild-to-moderate gastrointestinal-related TEAEs being the most commonly reported.

Our study limitations included a very limited enrollment of patients <18 or ≥65 years of age. Although our inclusion criteria permitted females ≥12 years of age, no one <18 years of age received ibrexafungerp. Furthermore, only five patients ≥65 years of age, combined from both VANISH studies, were included in the mITT population. Future studies should continue to maintain eligibility criteria for patients ≥12 years of age to evaluate the safety and efficacy of ibrexafungerp for VVC in the younger and older patient populations.

Our results are also limited by the exclusion of patients with uncontrolled diabetes mellitus (hemoglobin A1c ≥7), which resulted in ∼7% of patients with diabetes mellitus enrolled in the ibrexafungerp and placebo groups. In comparison, historical fluconazole studies have either excluded patients with diabetes mellitus^23^ or have treated only a small proportion of patients (4.5% [14 of 309]) with single-dose or two sequential doses of fluconazole.^24^ Because diabetes mellitus has been associated with VVC infection,^29,30^ future studies should evaluate the effectiveness of ibrexafungerp in patients with diabetes mellitus, regardless of how well their disease is controlled. A final limitation of our study was the lack of an active comparator. However, FDA industry guidance for the development of drugs for the treatment of VVC advised for the appropriate use of placebo-controlled studies in acute VVC. Future comparative studies are being considered.^17^

## Conclusions

VVC is a common infection that negatively impacts the quality of life of affected women and is associated with meaningful direct and indirect costs.^1–3,7,8^ After more than 20 years without new treatment choices, women and their health care providers now have a new oral therapeutic option. Ibrexafungerp, a first-in-class nonazole triterpenoid, provides a new safe and effective oral treatment for VVC with the convenience of 1-day dosing. This pooled analysis strengthens and confirms the efficacy and safety data presented in VANISH 303 and VANISH 306. Patient subgroup analyses suggest that race, BMI, baseline VSS scores, and *C albicans* infections do not adversely affect ibrexafungerp efficacy, thereby making ibrexafungerp an appropriate therapy for patients with acute VVC. Unlike current azole treatments for patients with severe VVC (VSS score ≥7) at baseline, which usually require >1 day of therapy, ibrexafungerp provides significant clinical cure rates with just 1 day of treatment.

## Data Sharing Statement

Qualified scientific and medical researchers may make requests for individual participant data that underlie the results (text, tables, and figures) reported in this article, after deidentification, at medicalaffairs@SCYNEXIS.com Methodologically sound proposals for such data will be evaluated and approved by SCYNEXIS, Inc., in its sole discretion. All approved researchers must sign a data access agreement before accessing the data. Data will be available as soon as possible but no later than within 1 year of the acceptance of the article for publication, and for 3 years following article publication. SCYNEXIS, Inc., will not share identified participant data or a data dictionary.
